# Decline in perception of acid regurgitation symptoms from gastroesophageal reflux disease in diabetes mellitus patients

**DOI:** 10.1371/journal.pone.0194466

**Published:** 2018-03-15

**Authors:** Kosuke Sakitani, Nobumi Suzuki, Sozaburo Ihara, Yoshihiro Hirata, Shoji Kawazu, Yasuhiko Iwamoto, Kazuhiko Koike

**Affiliations:** 1 The Institute for Adult Diseases, Asahi Life Foundation, Tokyo, Japan; 2 Graduate School of Medicine, the University of Tokyo, Department of Gastroenterology, Tokyo, Japan; Campus Bio Medico University, ITALY

## Abstract

**Objectives:**

To determine if a discrepancy exists between subjective symptoms and the grade of endoscopic gastroesophageal reflux disease (GERD) in diabetes mellitus (DM) patients.

**Methods:**

All 2,884 patients who underwent esophagogastroduodenoscopy completed the modified Gastrointestinal Symptom Rating Scale (GSRS), an interview-based rating scale consisting of 16 items including a question on acid regurgitation. Patients were divided into DM and non-DM groups (1,135 and 1,749 patients, respectively). GERD was diagnosed endoscopically and graded according to the Los Angeles classification. Grade B or more severe GERD was defined as severe endoscopic GERD. The intergroup GSRS score was compared statistically.

**Results:**

In severe endoscopic GERD patients, the prevalence of patients with a positive GSRS score in the acid regurgitation question was statistically lower in DM patients than non-DM patients. Of the 60 non-DM patients with severe endoscopic GERD, 40 patients (67%) had a positive GSRS score for acid regurgitation; however, of the 51 DM patients with severe endoscopic GERD, 23 patients (45%) had a positive GSRS score. Multivariate analysis showed that severe endoscopic GERD (OR: 2.01; 95% CI: 1.21–3.33; p = 0.0066), non-DM (OR: 0.74; 95% CI: 0.54–0.94; p = 0.0157), younger age (OR: 0.98; 95% CI: 0.97–0.99; p = 0.0125), and hiatal hernia (OR: 1.46; 95% CI: 1.12–1.90; p = 0.0042) were associated with acid regurgitation symptoms.

**Conclusions:**

There is a discrepancy between subjective symptoms and endoscopic GERD grade in DM patients. The ability of DM patients to feel acid regurgitation may be decreased.

## Introduction

The prevalence of diabetes mellitus (DM) is rapidly increasing and there is mounting evidence that DM enhances the risk of malignancies, including esophageal adenocarcinoma, which has a poor prognosis [1–4]. It has been reported that chronic inflammation triggered by gastric acid reflux into the esophagus or gastroesophageal reflux disease (GERD) can lead to Barrett’s esophagus, which is the main precancerous change in esophageal adenocarcinoma [5, 6]. Thus, it is critical to assess GERD because it may be the start of carcinogenesis, especially among patients at high risk of esophageal adenocarcinoma. GERD is diagnosed when mucosal changes are observed during endoscopy, and the Los Angeles (LA) classification is commonly used to grade the reflux esophagitis endoscopically [7, 8]. GERD is also perceived and diagnosed by subjective symptoms such as heartburn and acid regurgitation [9, 10]. DM patients can develop neuropathy and their ability to feel pain can decrease [11, 12]. Therefore, subjective symptoms of GERD may be underestimated in DM patients [13, 14]. To the best of our knowledge, research on GERD that combines subjective symptoms and endoscopic findings in DM patients is insufficient. We aimed to examine if a discrepancy exists between subjective symptoms and endoscopic GERD grade in DM patients.

## Methods

### Patients

From May 2015 to September 2017, patients who underwent esophagogastroduodenoscopy at our institution were consecutively enrolled (n = 3,368). We excluded patients who met the following criteria: i) those with a previous history of gastrectomy (n = 53), and ii) those receiving proton pump inhibitors (PPIs), a class of established medications used to treat GERD symptoms and mucosal damage (n = 431) [15]. The remaining 2,884 patients were analyzed. Patients were divided into DM and non-DM groups. DM was diagnosed according to the 2010 Japan Diabetes Society (JDS) criteria [16]. Expert nurses confirmed the patients’ blood test results, medications, and history of DM. The study design was approved by the Ethics Committee at the Institute for Adult Diseases, Asahi Life Foundation, and conformed to the Declaration of Helsinki. The patient records were anonymized prior to analysis. Written informed consent was obtained from all participants.

### Subjective symptoms

All of the patients completed the modified Gastrointestinal Symptom Rating Scale (GSRS), an interview-based rating scale originally consisting of 15 items including a question on acid regurgitation relating to the previous seven days [17]. We added a question on “pharyngeal discomfort.” Specifically, the patients answered questions on 16 items: 1) Abdominal pain, 2) Heart burn, 3) Acid regurgitation, 4) Sucking sensations in the epigastrium, 5) Nausea and vomiting, 6) Borborygmus, 7) Abdominal distention, 8) Eructation, 9) Pharyngeal discomfort, 10) Increased flatus, 11) Decreased passage of stool, 12) Increased passage of stool, 13) Loose stools, 14) Hard stools, 15) Urgent need for defecation, and 16) Feeling of incomplete evacuation. Questions on items 1–9 are related to upper gastrointestinal symptoms and questions on items 10–16 are related to lower gastrointestinal symptoms. The GSRS uses a seven-grade Likert-type scale (a score of 1 represents an absence of symptoms, and a score of 7 represents very bothersome symptoms) [17].

### Endoscopy

Experienced endoscopists performed upper gastrointestinal endoscopies. Esophageal hiatal hernia, GERD (modified LA classification: Grades M, A, B, C, and D, where Grade B or more severe GERD was defined as severe endoscopic GERD) [8], gastric atrophy (Kimura–Takemoto classification: none as grade 0, mild as grade 1 [C-I and C-II], moderate as grade 2 [C-III and O-I], and severe atrophic gastritis as grade 3 [O-II and O-III]) [18, 19], and fundic glands polyp [20] were diagnosed endoscopically. Prior to the endoscopic examination, expert nurses used the GSRS and interviewed patients to obtain information on medications and medical history.

### Statistical analysis

All statistical analyses were performed using JMP10 software (SAS Institute, Cary, NC, USA). Welch’s *t*-test was used to compare the means of continuous variables. Comparisons of nominal variables were conducted using the chi-squared test or Fisher’s exact test as appropriate. The Mann–Whitney *U* test was used to compare GSRS score in the DM and non-DM groups. Odds ratios (OR) with 95% confidence intervals (CI) were used as a measure of association and were adjusted by unconditional logistic regression models. The prevalence of patients with a positive GSRS score (i.e., ≥ 2) in the DM and non-DM groups was compared using the Pearson’s chi-squared test. A *p*-value of less than 0.05 was considered to indicate statistical significance.

## Results

### Baseline characteristics

The baseline characteristics of the patients are provided in Table 1. In total, 2884 patients (2,091 males and 793 females, mean age 60.9 years, and mean body mass index [BMI] 23.7 kg/m^2^) underwent endoscopy and were assessed using the GSRS. The 2,884 patients comprised 1,749 non-DM patients and 1,135 DM patients (55 type 1 DM and 1,080 type 2 DM). Non-DM patients were significantly younger than DM patients (56.6 and 67.5 years, respectively). The mean BMI was significantly higher in DM patients than non-DM patients (23.2 kg/m^2^ for non-DM patients and 24.1 kg/m^2^ for DM patients). DM patients had higher degrees of atrophic gastritis and endoscopic GERD, more hiatal hernia, and less gastric fundic polyps than non-DM patients.

**Table 1:** Demographic data of non-DM and DM patients.

Total (n = 2,884)	Non-DM (n = 1,749)	DM (n = 1,135)	*p*
Mean age (range), years	56.6 (21–91)	67.5 (28–90)	<0.0001*
Sex, n (%)			0.0027*
Female	516 (29.5%)	277 (24.4%)	
Male	1233 (70.5%)	858 (75.6%)	
Mean body mass index (range), kg/m^2^	23.2 (15.5–39.8)	24.1 (15.6–48.3)	<0.0001*
Atrophic gastritis grade, n (%)			<0.0001*
None: grade 0	917 (52.4%)	415 (36.6%)	
Mild: grade 1	329 (18.8%)	211 (18.6%)	
Moderate: grade 2	334 (19.1%)	241 (21.2%)	
Severe: grade 3	169 (9.66%)	268 (23.6%)	
Hiatal hernia, n (%)	300 (17.2%)	228 (20.1%)	0.046*
GERD, n (%)			0.031*
None	1272(72.7%)	843 (74.3%)	
Grade M	212 (12.1%)	101 (8.90%)	
Grade A	205 (11.7%)	140 (12.3%)	
Grade B	52 (2.97%)	38 (3.35%)	
Grade C	7 (0.40%)	12 (1.06%)	
Grade D	1 (0.057%)	1 (0.088%)	
Fundic glands polyp, n (%)	316 (18.1%)	158 (13.9%)	0.0027*

DM: diabetes mellitus, GERD: gastroesophageal reflux disease

*: *p*<0.05

### GSRS score in non-DM and DM patients

In non-DM and DM patients, the mean scores for each of the 16 items in the GSRS are shown in Table 2. In DM patients, the scores for items 11 (decreased passage of stool), 14 (hard stools), and 16 (feeling of incomplete evacuation) were higher than in non-DM patients. On the other hand, in non-DM patients, the scores for items 1 (abdominal pain), 2 (heart burn), 3 (acid regurgitation), 4 (sucking sensations in the epigastrium), 6 (borborygmus), 7 (abdominal distention), 8 (eructation), 9 (pharyngeal discomfort), and 12 (increased passage of stools) were higher than in DM patients.

**Table 2:** Mean GSRS score of non-DM and DM patients.

Total (n = 2,884)	Non-DM (n = 1,749)	DM (n = 1,135)	*p*
① Abdominal pains	1.55	1.37	<0.0001*
② Heart burn	1.50	1.44	0.0117*
③ Acid regurgitation	1.56	1.46	0.0010*
④ Sucking sensations in the epigastrium	1.42	1.30	<0.0001*
⑤ Nausea and vomiting	1.31	1.30	0.2523
⑥ Borborygmus	1.60	1.40	<0.0001*
⑦ Abdominal distention	1.58	1.48	0.0149*
⑧ Eructation	1.70	1.57	0.0006*
⑨ Pharyngeal discomfort	1.48	1.40	0.0014*
⑩ Increased flatus	1.95	2.03	0.1166
⑪ Decreased passages of stools	1.66	2.05	<0.0001*
⑫ Increased passage of stools	1.64	1.55	0.0138*
⑬ Loose stools	1.66	1.56	0.0086*
⑭ Hard stool	1.54	1.88	<0.0001*
⑮ Urgent need for defecation	1.60	1.66	0.1367
⑯ Feeling of incomplete evacuation	1.86	2.01	0.0001*

GSRS: Gastrointestinal Symptom Rating Scale, DM: diabetes mellitus

*: *p*<0.05

Next, we examined which of the 16 subjective symptoms could predict severe endoscopic GERD (LA classification B and more severe) in non-DM patients (Table 3). After adjusting for sex, age and BMI, the OR for severe endoscopic GERD was as high as 2.88 for item 3 (acid regurgitation). Questions on symptoms of the upper gastrointestinal tract other than item 3 and all questions related to the lower digestive tract were not related to endoscopic severe GERD.

**Table 3:** Associated factors for severe GERD of non-DM patients.

n = 1,749	Multivariate OR (95% CI)	*p*
Age (year)	0.98 (0.94–1.03)	0.0714
Male sex	4.32 (0.96–19.4)	0.0559
Body mass index (kg/m^2^)	1.12 (0.99–1.26)	0.0714
① Abdominal pains	1.62 (0.51–5.12)	0.4104
② Heart burn	0.76(0.23–2.48)	0.6597
③ Acid regurgitation	2.88 (1.00–8.31)	0.0493*
④ Sucking sensations in the epigastrium	0.90 (0.27–2.91)	0.8654
⑤ Nausea and vomiting	0.62 (0.17–2.25)	0.4738
⑥ Borborygmus	1.14 (0.40–3.27)	0.7973
⑦ Abdominal distention	0.63 (0.20–2.03)	0.4484
⑧ Eructation	0.97 (0.35–2.63)	0.9533
⑨ Pharyngeal discomfort	0.82 (0.25–2.69)	0.7526
⑩ Increased flatus	1.48 (0.48–1.53)	0.4887
⑪ Decreased passages of stools	2.46 (0.77–7.88)	0.1272
⑫ Increased passage of stools	1.00 (0.23–4.37)	0.9966
⑬ Loose stools	0.65 (0.15–2.94)	0.5805
⑭ Hard stool	0.49 (0.13–1.61)	0.2290
⑮ Urgent need for defecation	2.08 (0.66–6.57)	0.2087
⑯ Feeling of incomplete evacuation	1.44 (0.47–4.34)	0.5142

GERD: gastroesophageal reflux disease, DM: diabetes mellitus, OR: Odds ratio, CI: Confidence interval

*: *p*<0.05

### Severe GERD and GSRS score

**Fig 1:**
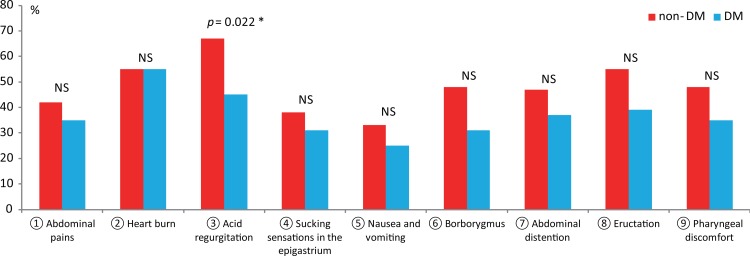
Percentage of positive GSRS scores (≥ 2) for items 1–9 in severe endoscopic GERD patients. The percentage of patients with GSRS scores ≥ 2 for items 1–9 in non-DM (n = 60) and DM (n = 51) patients with severe endoscopic GERD are shown.

Next, we examined the mean upper gastrointestinal GSRS score (items 1–9) in non-DM and DM patients with severe GERD. As shown in Fig 1, of the 60 non-DM patients with severe endoscopic GERD, 40 patients (67%) had a positive GSRS score for acid regurgitation; however, of the 51 DM patients with severe endoscopic GERD, 23 patients (45%) had a positive GSRS score. The prevalence of severe endoscopic GERD patients with a positive GSRS score was significantly lower in DM patients than in non-DM patients. Finally, we conducted a multivariate analysis to examine factors related to a positive score for item 3 (acid regurgitation). As shown in Table 4, in addition to severe endoscopic GERD (OR: 2.01; 95% CI: 1.21–3.33; *p* = 0.0066), non-DM (OR: 0.74; 95% CI: 0.54–0.94; p = 0.0157), younger age (OR: 0.98; 95% CI: 0.97–0.99; *p* = 0.0125), and hiatal hernia (OR: 1.46; 95% CI: 1.12–1.90, *p* = 0.0042) were associated with acid regurgitation symptoms.

**Table 4:** Associated factors for positive GSRS score of acid regurgitation.

n = 2,884	Multivariate OR (95% CI)	*P*
Age (year)	0.98 (0.97–0.99)	0.0125*
Male sex, yes	0.83 (0.65–1.07)	0.1574
Diabetes mellitus, yes	0.74 (0.54–0.94)	0.0157*
Body Mass Index (kg/m^2^)	1.02 (0.99–1.05)	0.6851
Atrophic gastritis (grade 0 to 3)	0.99 (0.89–1.10)	0.8846
Severe GERD, yes	2.01 (1.21–3.33)	0.0066*
Hiatal Hernia, yes	1.46 (1.12–1.90)	0.0042*
Fundic glands polyp, yes	0.99 (0.73–1.35)	0.9736

GSRS: Gastrointestinal Symptom Rating Scale, GERD: gastroesophageal reflux disease, OR: Odds ratio, CI: Confidence interval

*: *p*<0.05

## Discussion

In this study, we examined the relationship between subjective symptoms and endoscopic GERD in DM and non-DM patients. Our results indicate that the question on acid regurgitation in the GSRS is useful for detecting severe endoscopic GERD in patients with and without DM. Furthermore, even in patients with endoscopic severe GERD, the frequency of symptoms of acid regurgitation was lower in DM patients than non-DM patients. It is reported that endoscopic severity of GERD and subjective symptom do not necessarily correlate or weakly correlated [9, 21–23], and the degree of subjective symptoms declines in the elderly [24, 25]. The result that the younger age in this study appeals the symptoms of acid reflux (Table 4) agrees with the previous reports.

Several questionnaires have been evaluated for their ability to accurately identify GERD [26–28]. The GSRS, the questionnaire we used in this study, has also been used to assess GERD symptoms [29, 30]. Constipation is a common symptom in DM patients [31]. In line with this, our present analysis revealed that DM patients tended to have a higher score for items 11 (decreased passage of stool), 14 (hard stool), and 16 (feeling of incomplete evacuation) in the GSRS (Table 2). We believe that gastrointestinal motility impairment due to diabetic neuropathy may be a possible explanation [12]. By contrast, fewer complaints of acid regurgitation symptoms, which is a typical GERD symptom [32], in DM patients is thought that it may have been caused by perceptual decline due to diabetic neuropathy [12].

There were limitations to our study. First, this was a cross-sectional study; as such, a future prospective study is required. Second, pH monitoring, which is regarded as a gold standard method for diagnosing GERD [33], was not used in this study. Third, information that may have affected the results, such as the duration of DM, life styles of the patients, medications other than PPIs, and *Helicobacter pylori* infection status [18, 34], were not obtained in this study.

In conclusion, there is a decline in the perception of acid regurgitation symptoms associated with GERD in DM patients. It may be important to perform endoscopic work-up in DM patients to identify possible precancerous lesions even if these patients do not claim to have acid regurgitation symptoms.

## Abbreviations

DM: Diabetes mellitus
GERD: Gastroesophageal reflux diseases
GSRS: Gastrointestinal Symptom Rating Scale
PPIs: Proton pump inhibitors
